# Tick-Borne Flaviviruses and the Type I Interferon Response

**DOI:** 10.3390/v10070340

**Published:** 2018-06-21

**Authors:** Richard Lindqvist, Arunkumar Upadhyay, Anna K. Överby

**Affiliations:** 1 Department of Clinical Microbiology, Virology, Umeå University, SE-90185 Umeå, Sweden; richard.lindqvist@umu.se (R.L.); upadhyay.arunkumar@umu.se (A.U.); 2 Laboratory for Molecular Infection Medicine Sweden (MIMS), Umeå University, SE-90187 Umeå, Sweden; 3 Umeå Centre for Microbial Research (UCMR), Umeå University, SE-90187 Umeå, Sweden

**Keywords:** tick-borne flavivirus, innate immunity, interferon, tick-borne encephalitis virus, powassan virus, omsk hemorrhagic fever virus, kyasanur forest disease virus, louping ill virus, viperin

## Abstract

Flaviviruses are globally distributed pathogens causing millions of human infections every year. Flaviviruses are arthropod-borne viruses and are mainly transmitted by either ticks or mosquitoes. Mosquito-borne flaviviruses and their interactions with the innate immune response have been well-studied and reviewed extensively, thus this review will discuss tick-borne flaviviruses and their interactions with the host innate immune response.

## 1. Introduction

Tick-borne flaviviruses (TBFV), *Flaviviridae* family, includes many pathogens causing severe human disease, ranging from mild fever to encephalitis and hemorrhagic fever. There are more than 70 viruses in the genus flavivirus, and they are transmitted by arthropods such as mosquitoes (dengue virus (DENV), Japanese encephalitis virus (JEV) and West Nile virus (WNV), yellow fever virus (YFV), and Zika virus (ZIKV) and ticks (tick-borne encephalitis virus (TBEV), Langat virus (LGTV), Kyasanur forest disease virus (KFDV), Omsk hemorrhagic fever virus (OHFV), Powassan virus (POWV), and Louping-ill virus (LIV)) [1,2,3,4,5]. Among the TBFVs, TBEV, POWV, and LIV are encephalitic, whereas OHFV and KFDV are hemorrhagic viruses.

There are general features that distinguish TBFV from mosquito-borne. One such feature is that tick-borne viruses tend to persist in certain stable foci whereas mosquito-borne viruses may suddenly emerge and they can rapidly spread to new areas and continents causing large epidemics [6,7,8,9,10,11]. Mosquito-borne flaviviruses are transmitted horizontally from mosquito to vertebrate to mosquito, and for some viruses, humans act as the amplifying host [12,13,14]. Mosquitoes have a short life compared to ticks, as it can take several years for a tick to develop from egg to adult [15]. Ticks also only take one blood meal at each of their life stages, larvae, nymph, and adult, this means that tick-borne viruses need to be maintained in infected ticks during a very long time [16]. Thus, replication in ticks needs to be lower compared to mosquitoes, which have higher viral turnover and a faster generation cycle, this also contributes to the high mutation rate in mosquito-borne flaviviruses, and relative stable genomes of TBFVs [17,18]. Infection of tick-borne flaviviruses usually cause a short viremia in humans and larger vertebrates. This route of transmission has been described as less important in tick-borne flavivirus infection [19], instead TBEV has been shown to be transmitted by viremic rodents and among co-feeding ticks without viremia [20]. Humans act as dead-end hosts since they do not produce high enough viremia to transmit the virus to new ticks.

### 1.1. Tick-Borne Encephalitis Virus

**Figure 1:**
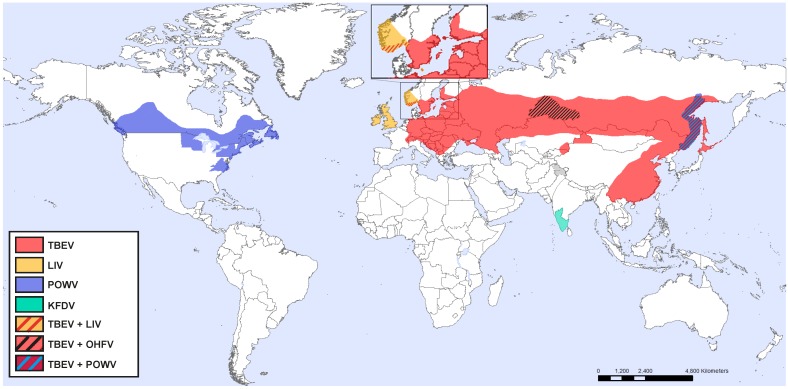
Approximate distribution of TBEV, LIV, POWV, KFDV, and OHFV.

According to phylogenetic differences, TBEV has been divided into three different subtypes, European, Siberian, and Far Eastern. The European subtype is mainly transmitted by *Ixodes ricinus*, whereas the Siberian and Far Eastern subtypes are primarily transmitted by *Ixodes persulcatus* [21,22]. TBEV is found in central, eastern, and northern Europe and Asia (Figure 1) and correlates with the presence of infected ticks. *Ixodes ricinus* is found throughout Europe, whereas *Ixodes persulcatus* is found in Eastern Europe in the west, and China and Japan in the east [23].

TBEV is considered one of the most important arboviruses in central and eastern European countries and in Russia, with about 13,000 estimated human cases annually [24]. In fact, over the last decade there has been an approximately 300% increase in the number of TBE cases in Europe [25], and TBEV is currently spreading into new regions in France, Sweden, Norway, and Italy [26,27,28,29]. This increase is thought to be due to growth in population and spread of ticks, which is promoted by factors including climate change, social and political change, and changes in the land use [30,31]. The increased expansion in Europe also poses an increased risk for the population engaged in outdoor activities.

TBEV is a zoonotic disease and the natural cycle of TBEV is dependent and maintained in a complex cycle involving ticks as the vector and reservoir of the virus and small rodents as hosts for ticks [32]. Humans are not part of the natural transmission cycle of TBEV and are the incidental host when infected by a bite from an infected tick [33]. Transmission through consumption of unpasteurized milk has also been reported for TBEV [34,35], as well as transmission via solid organ transplant [36].

During the tick bite, the virus is inoculated into the skin of the vertebrate host. The initial replication is believed to occur locally in the dendritic cells. This is followed by infection of the draining lymph nodes, resulting in the primary viremia and subsequent infection of the peripheral tissues, where further replication maintains the viremia for several days [37,38,39,40]. The disease course of TBEV is biphasic; the initial phase is characterized by flu-like symptoms and is followed by a second phase involving CNS infection, with meningitis, encephalitis, or meningoencephalitis [41,42,43]. The mortality rate of TBEV varies from 1 to 20% depending on the subtype, in which the European TBEV subtype has shown lower mortality rates compared to the Siberian and Far Eastern [23,40,44]. Among the patients that experience neuroinvasive TBEV infection, approximately 25–40% of the survivors suffer from long lasting neurological sequelae [45,46]. No antivirals are available for treatment of TBEV infection but there is an effective vaccine [25,47].

LGTV, which is closely related to TBEV, is found in south east Asia and Russia [17]. LGTV has not been associated with human disease under natural infections although it shares 84% sequence identity with TBEV [17]. Because of its avirulence in humans and close similarity to TBEV, LGTV is often used as a model virus for TBEV under biosafety level-2 conditions.

### 1.2. Powassan Virus

POWV is found in Russia and North America, and is the only TBFV present in America (Figure 1) [48]. It is transmitted by *Ixodes scapularis*, *Ixodes cookei*, and several other *Ixodes* tick species, to small and medium size mammals, whereas humans are accidental dead-end hosts. Milk-borne POWV transmission might also be possible since POWV virus has been found to be secreted in milk under experimental settings [49].

Although not much is known about POWV pathogenesis, recent studies in mice have found that tick saliva was important to enhance POWV transmission and the outcome of disease [50]. Furthermore, it has been demonstrated that POWV infects macrophages and fibroblasts in the skin, shortly after the tick bite, also, other unidentified cells were shown to be infected [51]. Interestingly, macrophages were found to be the primary target for POWV in the spleen [52], and in the CNS, which is the main target site for POWV infection, neurons have been shown to be the primary target for POWV in mice and humans [52,53].

During the last 10 years there has been an increase of POWV in the USA with approximately 100 reported cases [54,55]. The recent rise in incidence could be due to increased surveillance and diagnosis of POWV, or it may represent a true emergence of the disease in endemic areas, or both [55]. The incubation period ranges from 1 week to 1 month. The symptoms of POWV infection may include fever, headache, vomiting, weakness, confusion, seizures, and memory loss with a case fatality rate of 10% [54]. Approximately half of the survivors experience permanent neurological symptoms, such as recurrent headaches, muscle wasting, and memory problems (https://www. CDC.gov). There are no antiviral treatments or vaccines available against POWV.

### 1.3. Louping-Ill Virus

LIV is mainly distributed in the UK and Ireland, but it has also been detected in sheep in Norway and on the Bornholm island in Denmark (Figure 1). LIV is most commonly known as pathogen of sheep and red grouse although humans can also get infected [17,56]. Animals develop a febrile disease, which can progress to fatal encephalitis [57]. Like TBEV, the vector of LIV is the tick species *Ixodes ricinus* [57]. Ticks transmit the virus to animals, however, natural exposure to humans is rare [33]. Instead humans that are exposed to infected animals such as veterinarians, farmers, butchers, and abattoir workers, as well as laboratory scientists have acquired LIV infection [58,59,60,61]. In between the years of 1934 and 1991, 31 human cases of LIV was described [62]. In humans, LIV causes a disease that closely resembles TBEV, with initial flu-like symptoms which can progress to severe neurological disease. Distinct but closely related viruses are also found in Spain (Spanish sheep encephalomyelitis virus), Turkey (Turkish sheep encephalitis virus), and Greece (Greek goat encephalitis virus) [33].

### 1.4. Omsk Hemorrhagic Fever Virus

OHFV distribution is restricted to western Siberia (Figure 1) [17]. The main vector of OHFV is the meadow tick, *Dermacentor reticulatus*, which can also transmit the virus to humans. However, humans are mainly infected after contact with infected muskrats (*Ondatra zibethicus*) which are very sensitive to the infection and often succumb to the infection [63]. Muskrats develop high viremia which can last for several weeks. Human infection occurs through contact with urine, feces, and blood [63]. Secretion of OHFV in unpasteurized goat milk has been reported but no milk-borne outbreaks have been observed [63]. The exact number of annual cases are uncertain because of misdiagnoses and unreported cases, but 165 cases were reported between 1988 and 1997 [63]. OHFV may cause a biphasic disease; the initial phase is characterized by high fever, bleeding from the nose, mouth, and uterus. Thirty to fifty percent of the cases experience a second phase characterized by high fever and reappearance of the symptoms from the initial phase. Case fatality rates range from 0.5 to 2.5% [63]. No antiviral treatments are available against OHFV, instead treatment is focused on supportive care to minimize hemorrhage and other complications [17].

### 1.5. Kyasanur Forest Disease Virus

KFDV is only found in India (Figure 1), although a similar genetic variant, Alkhurma hemorrhagic fever virus (AHFV) has been detected in Saudi Arabia [17,47]. KFDV is mainly transmitted by ticks belonging to the genus *Hemaphysalis* but other tick genera have also been shown to be able to transmit KFDV [64,65]. During hemorrhagic KFDV infection, the initial phase is similar to TBEV infection with fever and flu-like symptoms, but it may also include bleeding from the nose, mouth or gastrointestinal tract [65,66]. The second phase, which is experienced by 1–20% of the cases, includes neurological symptoms such as headache, mental disturbance, and tremor, however, no evidence of meninges or encephalitis have been found [65]. There are about 400–500 reported annual cases of KFDV [47], with case fatality rates ranging from 3 to 10% [17,65]. In 1990 a KFDV vaccine was developed, although it provided some protection, due to low efficacy, the number of cases still increased from 1999 to 2012 [67,68,69]. Treatment of KFDV is limited to supportive care [47].

## 2. TBFV Genomic Organization

TBFVs are enveloped viruses around 50 nm in diameter. The envelope carries two surface proteins, the envelope (E) protein and the membrane (M) protein. The latter is derived from a precursor protein, prM. The nucleocapsid (NC) lies inside the viral envelope and consists of multiple copies of capsid (C) protein and the viral genome. The TBFV genomes are single stranded, positive-sense RNA of approximately 11,000 nucleotides. It has a 5′-cap with a single open reading frame (ORF). The ORF is flanked by 5′ and 3′ untranslated regions (UTRs). The viral protein is encoded by the ORF as a single polyprotein, which is co- and post-translationally cleaved by cellular and viral proteases into individual viral proteins (three structural and seven non-structural). The polyprotein is arranged in the order 5′-C-prM-E-NS1-NS2A-NS2B-NS3-NS4A-NS4B-NS5-3′ [70,71].

## 3. Replication Cycle of TBFV

**Figure 2:**
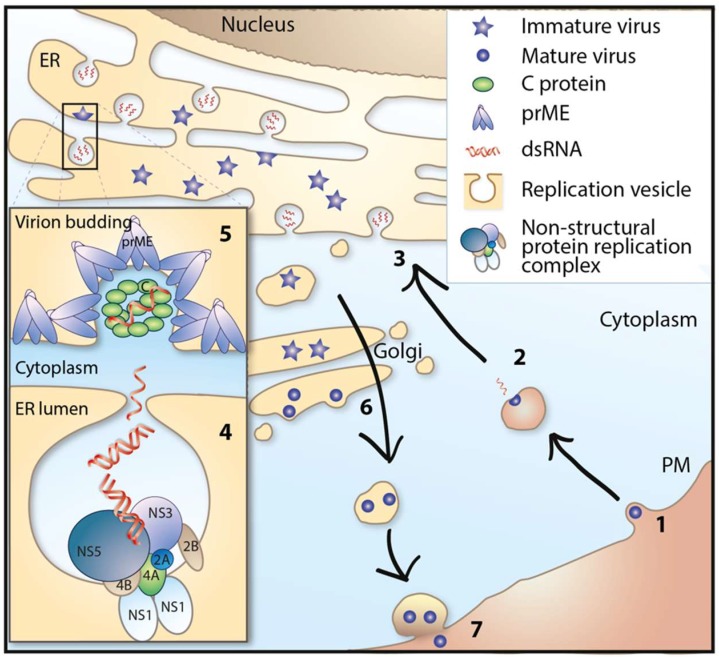
Life cycle of tick-borne flaviviruses. (1) Receptor binding and endocytosis. (2) Membrane fusion and release of viral genome. (3) Translation of viral RNA and synthesis of negative strand RNA from plus strand viral RNA template. (4) Genome replication. (5) Genome packaging and assembly of virions. (6) Maturation of virions, furin cleavage, and transport via the secretory pathway. (7) Release of virus from the cell. ER = endoplasmic reticulum, PM = plasma membrane.

The first step of virus replication starts when the virus binds to its receptor and is taken up into the cell by receptor-mediated endocytosis. The attachment is mediated by viral E protein and entry receptor on the host cell. The entry receptor for TBFVs has not been identified, but attachment to heparan sulphate and glycosaminoglycan, which are present in abundance on many cell types of both vertebrates and ticks, is predicted to play a role during binding and entry [72]. Once the virus has entered the cell, it is transported to endosomes, where the acidic environment of these vesicles leads to reorganization and conformational change of the E protein, resulting in the fusion of the viral and endosomal membrane and the release of the viral capsid into the cytoplasm [73,74,75]. The viral RNA (vRNA) also functions as mRNA, associating with ribosomes to produce the polyprotein. The transmembrane domain of the viral protein is recognized as a signal peptide and recruits the vRNA/ribosomes/nascent polypeptide complex to the ER membrane, where it is co-translationally translocated into the ER membrane [76,77]. The nascent polypeptide is then processed by the cellular and viral proteases into structural and non-structural (NS) viral proteins. Some of the viral proteins such as NS2B, NS4A, and NS4B integrate and alter the membrane of the ER to form a membrane vesicular structure, with a small pore connecting the interior of the vesicle to the cytoplasm [78,79,80,81,82]. These vesicles are the site for the formation of replication complexes (RC) and RNA replication [80,82,83]. Following RNA replication, genomic RNA is proposed to exit the vesicle through the vesicular pore and is packaged by C proteins into the NC on the cytoplasmic side of the ER membrane [82,84,85]. During the process of budding of NC from the cytoplasm into the ER, it acquires the lipid envelope along with E and prM proteins, which are associated with the ER membrane. The mechanism that ensures efficient incorporation of NC into the ER membrane with the E and prM protein is poorly understood. Following budding into the ER lumen, the immature virions are transported through the cellular secretory pathways in a COPI and COPII dependent manner [86] to the extracellular medium. The immature virion has heterodimers of E and prM that completely cover the lipid bilayer to form a spiky proteinaceous coat. During the transport through the Golgi, the E protein is glycosylated [87]. The acidic environment of the Golgi induces conformational changes in the E and prM proteins, which exposes a cleavage site on prM. Cleavage of prM by the cellular protease furin results in the formation of a mature virion [88,89]. Mature virions are then released by exocytosis, which completes the viral life cycle [90,91,92] (Figure 2).

## 4. Tick–Virus Interactions

In arthropods, such as mosquitos and ticks, RNA interferences (RNAi) are the most important antiviral defense and tick cells have been shown to mount RNAi responses against LGTV and TBEV [93]. Proteins involved in the RNAi mediated response such as Argonaute 30 (Ago30), Ago 16 and Dicer (Dcr) 90 were subsequently identified as inhibitors of LGTV [93]. Furthermore RNA-seq and mass spectrometric analysis revealed that when challenged by TBEV infection, tick cells upregulated genes involved in immunity and metabolism, whereas genes involved in cellular stress were downregulated [94]. Using gene silencing approaches, this study confirmed the antiviral effect of Ago 30 and Dcr 90 in tick cells. Furthermore, novel antiviral genes such as complement factor H, heat shock protein (HSP) 70 and 90 and trypsin was found to inhibit LGTV in tick cells [94]. Taken together, studies so far have identified activation and antiviral actions of the RNAi mediated response in tick cells as well as other inhibitory proteins such as HSP70, HSP90, and trypsin.

## 5. The Innate Immune Response

The innate immune response provides the first line of defense against viral infections. This defense includes physical and chemical barriers such as the skin and mucous membranes and the acidity of the stomach. They serve as an initial barrier that protects the host from infection. Once these barriers have been breached, the innate immune response relies on a set of germline-encoded receptors, known as pathogen recognition receptors (PRRs), which recognize pathogen specific molecular patterns that are sensed as a danger signal by the host cell [95,96]. Engagement of PRRs results in the activation of several defense mechanisms including, production of interferon (IFN), induction of phagocytosis, cytokines, chemokines, antimicrobial peptides, antiviral proteins, as well as activation of leukocytes and T-cells. Furthermore, the innate immune response harbors cellular components, such as dendritic cells, macrophages, and natural killer (NK) cells [97].

### 5.1. The Type I IFN Response

In 1957 Isaacs and Lindenmann discovered IFN as a secreted factor that interfered with viral replication [98]. In the early pioneer work on IFN in the fifties and sixties, TBEV served as a model system, and TBEV was shown to induce IFN after infection and was also sensitive to pretreatment of IFNs [80,99,100,101,102]. Pretreatment of IFN was also found to inhibit KFDV, OHFV, and POWV titers in A549 cells (adenocarcinomic human alveolar basal epithelial cells) [103], thus IFN pretreatment induced broad spectrum inhibition of TBFVs. IFN has also been found to be strongly induced in the brains of POWV infected *Peromyscus lecuopus*, which are known as a natural host of the virus [104]. Similarly, IFN was found to be induced in sheep infected with LIV [105], and IFN-stimulated genes were induced in brains of KFDV infected mice, indicating an activated IFN response [106].

Three distinct classes of IFN, type I (IFN α and β), type II (IFNγ), and type III IFN (IFNλ) have been described. The expression of the IFNλ-receptors and IFNγ are restricted, but the type I IFNs can be expressed by most cell types and their receptor, the interferon-α/β receptor (IFNAR) is expressed on all nucleated cells [107]. There are 17 different type I IFNs in humans; these cytokines are produced by cells upon recognition of pathogen-associated molecular patterns, which are foreign to the host cell [95,108], and mediates antiviral activity by autocrine and paracrine signaling through IFNAR [109,110]. Signaling through IFNAR induces an antiviral state by the expression of hundreds of interferon stimulated genes (ISGs) [111,112].

#### 5.1.1. The Role of RIG-I-Like and Toll-Like Receptor Signaling

The host cell detects invading pathogens through PRRs, which recognize foreign molecular patterns that are generated during infection. The particular PRR involved in the recognition of the pathogen depends on the infecting virus [108]. In flavivirus infection, the most important PRRs are Toll-like receptor (TLR)3, TLR7, TLR8, retinoic acid-inducible gene I (RIG-I), and melanoma differentiation-associated protein-5 (MDA-5). TLR3 recognizes double stranded (ds) RNA, which is formed as an intermediate during flavivirus replication [113,114]. TLR7 and 8, which are located in the endosomes, recognize single stranded (ss) RNAs, such as the genomic RNA of flaviviruses [95,115,116,117]. RIG-I and MDA-5 recognize viral RNA in the cytoplasm of infected cells; RIG-I recognizes short dsRNA and 5′ triphosphorylated and 5′ diphosphorylated ssRNA, whereas MDA-5 recognizes long dsRNA [118,119,120].

Upon recognition, the different PRR will recruit distinct adaptor molecules. TLR3 recruits TIR-domain-containing adapter-inducing interferon-β (TRIF) [121], TLR7 and 8 recruit myeloid differentiation primary response 88 (Myd88) [122], whereas the RIG-I-like helicases, RIG-I and MDA-5, recruit interferon-beta promoter stimulator-1 (IPS-1) (also known as mitochondrial antiviral-signaling protein (MAVS), virus-induced signaling adapter (VISA) and CARD adapter-inducing IFN-beta (Cardif)) [123,124,125,126]. Ligation of PRRs and downstream recruitment of the adapter molecules result in the activation of transcription factors NF-κB and interferon regulatory factor (IRF) 3 and IRF7, which translocate to the nucleus and induces the expression and subsequent secretion of type I IFN [127].

Although the innate antiviral response has been well-studied in mosquito-borne flavivirus infection, the responses to TBFVs are less investigated, and most studies have used TBEV which therefore will be the main focus of the remainder of this review.

In TBEV infection, IFNβ induction has been shown to correlate with amount of intracellular viral RNA, and the IPS-1 pathway has been shown to protect mice from lethal LGTV infection and prolong survival after TBEV infection [80,128]. Absence of IPS-1 resulted in a lower systemic IFNα response, which correlated with higher viral replication in the peripheral tissues [128]. Furthermore, IPS-1 was shown to be of particular importance for the IFN production locally within the brain, where IPS-1^−/−^ mice had lower induction of IFNβ within the olfactory bulb despite higher viral burdens [128]. Furthermore, IFNβ induction was shown to be completely dependent on IPS-1 and IRF3 in mouse embryonic fibroblasts and viral recognition through the IPS-1-pathway was shown to be dependent on RIG-I and not MDA-5 in human osteosarcoma cells (U2OS) [80,83]. Interestingly, RIG-I has been shown to co-localize with stress granules (SG) during TBEV infection [129]. SG contains ribonucleoprotein aggregates with translationally stalled mRNAs, 40S ribosomes, and several RNA-binding proteins. SG function to prevent the generation of defective proteins [130]. SG are induced during TBEV infection and SG components TIA-1/TIAR was found to bind viral RNA and inhibit viral translation [131]. Thus, SGs could serve as a platform for IFN induction during TBEV infection.

Little is known about the role of the TLR7/8-Myd88 pathway in tick-borne flavivirus infection; however, TLR7 was shown to suppress LGTV replication within neurons of the brain although it did not affect pathogenesis [132]. What we are aware of; no animal experiments have shown any significance of the TLR3-TRIF pathway in tick-borne flavivirus infection. However, several studies have looked at the prevalence of polymorphisms in the TLR3 gene in TBE patients [133,134,135,136]. In particular, one polymorphism has been investigated the T allele in rs3775291. This polymorphism has been shown to reduce TLR3 signaling by about 30% [137]. Although the data regarding TLR3 is somewhat conflicting, Grygorczuk et al. hypothesized that a functional TLR3 facilitates the onset of neurological disease [135] by supporting the penetration through the blood brain barrier, but has a protective effect during the established CNS infection [134]. The differences between studies might also be connected to the different subtypes of the TBEV strain and the genetic background of the studied populations [134].

Taken together, studies so far have demonstrated the importance of the RIG-I-like-IPS-1 pathway in tick-borne flavivirus infection, whereas the role of the TLR pathways remains unclear.

#### 5.1.2. Interferon Signaling and the ISG Response

After secretion, type I IFN signals in an autocrine and paracrine manner by binding to the heterodimeric IFNAR, which consists of two subunits, IFNAR1 and IFNAR2 [138]. These subunits are associated with Janus activated kinases (JAK) localized in the cytoplasm; IFNAR1 interacts with tyrosine kinase-2 (Tyk2), whereas IFNAR2 interacts with JAK1. Ligation of IFNAR results in activation of Tyk2 and JAK1, which subsequently phosphorylate signal transducers and activators of transcription (STAT)-1 and STAT-2. Phosphorylated STAT-1 and STAT-2 then form a heterodimer, which associates with IRF9 to form IFN-stimulated gene factor-3 (ISGF3) that binds to the IFN-stimulated response element (ISRE) in the promoter of many ISGs to enhance the transcription of several hundreds of IFN-stimulated genes [110,139,140]. Together, these ISGs act to coordinate an antiviral response that is able to inhibit almost any step in the viral life cycle [140].

The IFN response in vivo in mice is very important to protect mice from lethal infection with LGTV. Mice lacking IFNAR all succumbed within 6 days of infection, whereas 80% of WT mice (6–8-week-old) survived the infection. Interestingly, all IFNβ^−/−^ mice survived the infection, demonstrating that the IFNαs can compensate for loss of IFNβ in LGTV infection. Furthermore, IFNAR was shown to be a critical determinant of LGTV tropism as LGTV RNA was found in all organs in the absence of IFNAR, whereas in WT, only low viral burdens can be detected in the olfactory bulb [99,128,141]. Using transgenic mice, it was further shown that the IFN response was needed both in the peripheral tissue, as well as locally in the CNS, in order to clear LGTV infection [99,100].

Within the CNS, LGTV mainly infected neurons [99,128], which is also the case in lethal TBEV infection of humans [142]. Although neurons remain the main target cell of TBEV, astrogliosis have been shown in post mortem human brains [142,143]. In vitro studies on primary cortical astrocytes show a fast up regulation and secretion of IFN after TBEV infection, which is able to protect neighboring astrocytes and neurons from infection already 6 and 3 h post infection, respectively [100]. Astrocytes have been shown to be resistant to TBEV-induced cytopathic effects [144,145], and it was later shown that IFNAR expression protected the astrocytes from the virus-induced cytopathic effects [100]. Pretreatment of cells with IFNs strongly inhibits growth of most TBFVs in cell culture [100,103,146,147,148,149] and this is due to the upregulation and concerted actions of several hundreds of ISGs. Only a few ISGs have been identified to play a role in TBEV and LGTV infection [86,141,147,149,150]. One of them, the 2′-5′-oligoadenylate synthetase (2′-5′-OAS) (OAS) is activated by double-stranded RNA, leading to the OAS protein polymerization into 2′-5′-linked oligoadenylates (2-5As) [151,152]. These 2-5As activate RNase L, resulting in the degradation of viral RNA [153]. Several polymorphisms in the OAS genes have been shown to correlate with severe forms of TBE in patients [154]. Also, the murine isoform *Oas1b*, which is often lacking in inbred mice strains, confers strain dependent resistance against neurovirulence from far eastern TBEV [155]. Two other ISGs which have been shown to be antivirally active against TBEV are virus inhibitory protein endoplasmic reticulum associated interferon inducible (viperin) and tripartite motif-79α (TRIM79α).

The TRIM proteins are a family of proteins able to mediate antiviral activity against many different viruses [156]. The rodent specific TRIM79α was identified to interact with LGTV NS5 in a yeast two-hybrid screen. TRIM79α expression inhibited viral infection of LGTV and TBEV by mediating lysosomal dependent degradation of NS5. Interestingly this mechanism was quite specific to tick-borne flaviviruses as the mosquito borne WNV was not inhibited by TRIM79α nor did TRIM79α interact with NS5 of WNV [147].

**Figure 3:**
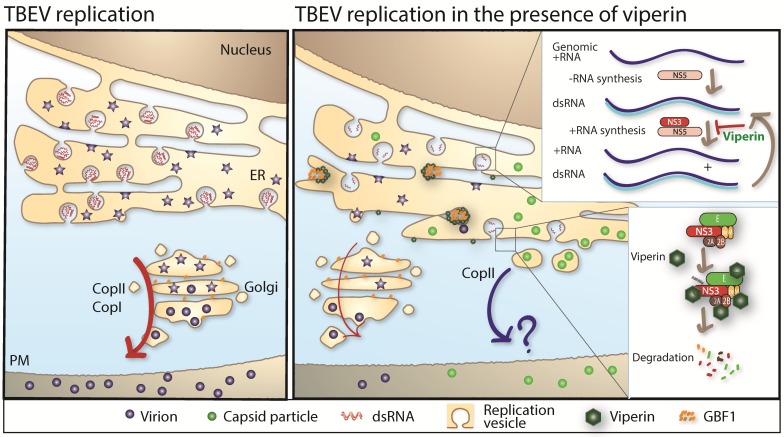
(**Left**) panel: in the absence of viperin, TBEV replicates efficiently inside membrane vesicular structures in the ER. Assembled virions are secreted through the secretory pathway in a COPII and COPI dependent manner. (**Right**) panel: in the presence of viperin, TBEV replication is strongly reduced by viperin. Viperin targets the NS3 protein for proteasomal degradation which inhibits the synthesis of + strand RNA. Furthermore, NS3 interacts with E, NS2A, and NS2B and these proteins are degraded by viperin in a NS3-dependent manner. Viperin also interferes with particle assembly by inducing secretion of C particles in a COPII-dependent manner, independent of COPI. Viperin mediates this effect by interacting and sequestering GBF1. Red arrow = secretory pathway, blue arrow = “?” vesicular transport via unknown pathway.

Viperin is highly conserved in evolution and was first identified as an IFN-inducible protein with antiviral activity against human cytomegalovirus in 2001 [157]. Over the last 17 years, viperin has gained lot of attention and was shown to exhibit broad antiviral activity [158]. Viperin is also known as one of the most highly upregulated genes after viral infection [146,159]. Within the family of *flaviviridae*, viperin has been shown to inhibit several members, such as WNV [160], DENV [161], ZIKV [162], hepatitis C virus [163], and TBEV [86,141,149,150]. Viperin is an iron sulphur protein with three domains; a N-terminus amphipathic alpha-helix which mediates the intracellular localization to the ER, a S-adenosylmethionine (SAM) radical domain [158,164,165] homologous to a family of proteins that use SAM as a cofactor [166], and a highly conserved C-terminal domain, important for iron sulphur (Fe/S) maturation [149,167]. Although viperin is able to inhibit several different viruses, the mechanism of action, the important motif in viperin and the step of viral life cycle inhibited differs for different viruses [158]. Viperin interacts with many viral and host factors for its antiviral function. TBEV replication is strongly inhibited by viperin, our research shows that viperin has no effect on the binding or entry of TBEV. However, viperin targets genome replication, packaging, and release of TBEV (Figure 3) [86,149,150]. Viperin specifically targets the plus-sense RNA synthesis with no significant effect on the negative-sense RNA of TBEV during genomic replication [149]. Viperin’s Fe/S maturation is dependent on Ciao1 [149,167]. The Fe/S cluster and a functional SAM domain of viperin is essential to inhibit the synthesis of the plus-sense RNA of TBEV [149]. However, the target for the radical SAM activity important for the antiviral activity of TBEV is currently unknown. The structure of viperin was recently characterized [168], and based on similarities to other radical SAM enzymes, several different hypothesis have been put forward regarding the substrate of viperin and its antiviral activity [168,169,170]. However, none of these have shown importance in the context of mammalian viral infection. Although the exact mode of action against TBEV is not well understood, recent data indicates that viperin interacts with several viral proteins; both structural prM and E and non-structural NS2A, NS2B, and NS3. These interactions lead to a viperin-NS3 dependent degradation of viral proteins. The degradation of TBEV NS3 was shown to be proteasome dependent [150] (Figure 3). Interestingly, IFN treatment induces an increase of TBEV capsid particles and this effect was found to be dependent on viperin. Viperin mediated this effect by interacting, via its N-terminus, with the cellular protein Golgi Brefeldin A resistant guanine nucleotide exchange factor 1 (GBF1) [86]. GBF1 is a key protein in the cellular secretory pathway and essential in the life cycle of many RNA viruses, which utilize vesicular trafficking in their replication cycle and assembly process [171,172,173,174] (Figure 3).

Although viperin has been demonstrated to be antiviral active against many different viruses in vitro, few studies have investigated viperin’s role in vivo. In TBFV infection, viperin was shown to control LGTV dissemination and replication in the brain after intraperitoneal administration, and viperin promoted survival after intracranial infection [141]. Interestingly, viperin has been shown to be expressed in the brain at the basal state [128], with high basal expression in primary astrocytes [100,141]. Viperin’s role within the brain during neurotropic LGTV infection was further mapped to certain brain regions, as viperin inhibited viral replication in the olfactory bulb and cerebrum, but not in the cerebellum or brainstem. This correlates very well with TBEV infection since viperin strongly inhibited TBEV replication in primary neurons and astrocytes from the cerebrum, but not in granular cell neurons isolated from the cerebellum. Interestingly, cortical neurons were completely dependent on viperin for IFN-mediated inhibition of TBEV, whereas in astrocytes, in which the IFN-mediated antiviral activities are strongly dependent on viperin, other ISGs could partly compensate for loss of viperin [141].

Taken together, viperin has been shown to be an ISG that strongly inhibits tick-borne flaviviruses in vivo and in vitro. This strong antiviral effect on TBEV is mediated by targeting the virus at multiple steps of the life cycle. Interestingly, viperin targeting the synthesis of plus-sense RNA is dependent on the SAM domain or the C-terminal domain, while the N-terminal domain of viperin is responsible for interfering with virus assembly and release.

#### 5.1.3. TBFV: Evasion and Antagonism of the Innate Immune Response

**Figure 4:**
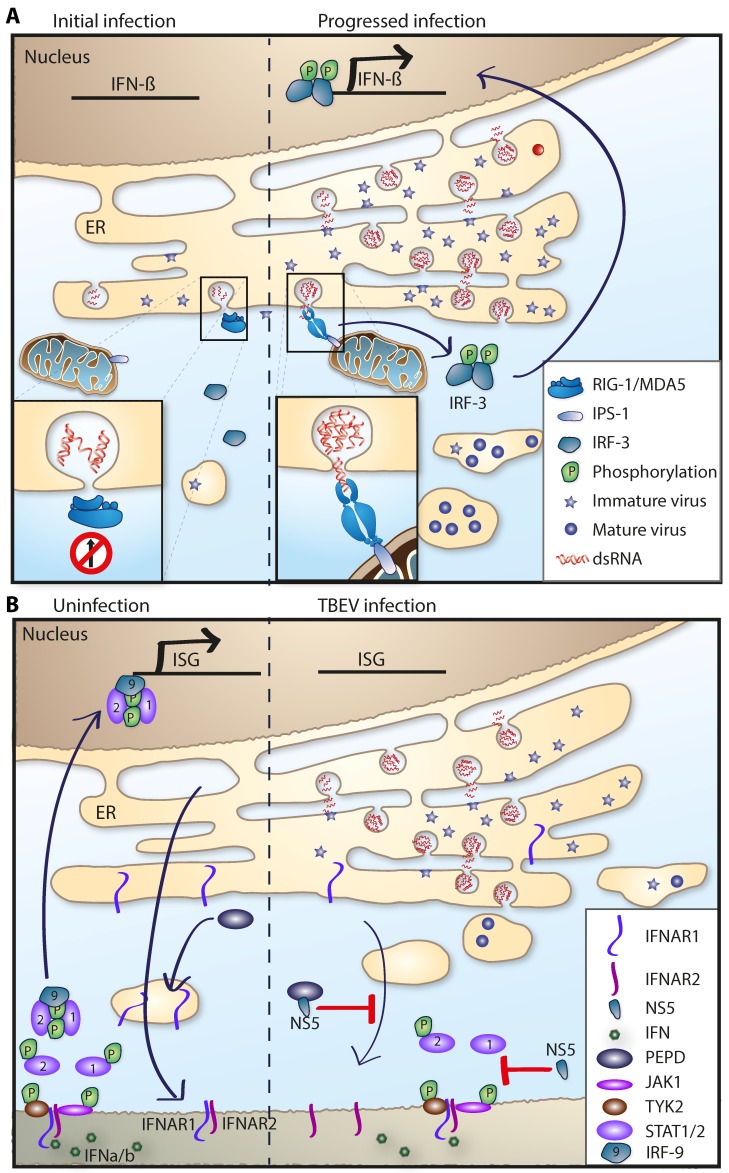
IFN evasion strategies of TBFV. (**A**) Passive evasion strategy. Viral NS proteins rearrange the ER membrane and induce replication vesicles or membrane packets. Viral replication and dsRNA is hidden away inside these vesicles as a passive way to prevent PPR recognition (red circle) and upregulation of IFNs. Later in infection, dsRNA leaks out and IFNs are upregulated via IRF3 and IPS-1/MAVS. (**B**) Left panel: PEPD is needed for maturation and subsequent transport of IFNAR1 to the plasma membrane. IFNAR1 and IFNAR2 heterodimer on plasma membrane can be activated by IFNα/β which leads to the signaling cascade and phosphorylation and translocation of STAT1/2-IRF9 into the nucleus and upregulation of ISGs. Right panel: NS5 interferes with IFN signaling. NS5 protein interacts with PEPD thus preventing IFNAR1 plasma membrane localization (red T). NS5 also prevents STAT1 phosphorylation (red T). Arrows indicate protein transport.

In order to establish an infection, a pathogen needs to overcome or evade the innate immune response. To breach the very first line of defense, the skin—TBFVs use the tick to deliver the virus through the skin via tick saliva. Furthermore, tick saliva contains immunomodulatory compounds that enhance viral transmission and dissemination [175,176]. Although there are several mechanisms in which mosquito-borne flaviviruses actively suppress the induction of type I IFN, no such mechanism has been identified in tick-borne flaviviruses so far [177]. Instead, TBEV, like other flaviviruses, utilizes a passive evasion mechanism in which the virus hides its dsRNA intermediates in vesicular structures inside the ER membranes, and thus delaying the recognition by the cytosolic RIG-I like receptors and subsequent IRF3 phosphorylation and IFN induction (Figure 4A) [80,81,83].

The most conserved inhibition of the type I IFN system within the *Flaviviridae* family is the antagonism of IFNAR signaling carried out by NS5; this mechanism is conserved between several mosquito and tick-borne flaviviruses [148,177,178,179,180,181]. In LGTV infection, NS5 was shown to inhibit the JAK-STAT pathway and NS5 was shown to interact with the IFNAR receptor [148]. Similarly, it was shown that NS5 of TBEV interacts with scribble (hScrib) which mediates NS5 localization to the plasma membrane and this interaction enables NS5 to inhibit type I and type II IFN mediated JAK-STAT signaling [178]. Knockdown of hScrib altered NS5 cellular localization and reversed the inhibition of the JAK-STAT signaling [178]. Further studies revealed that NS5 of TBEV inhibited the cell surface expression of IFNAR1 by binding to prolidase (PEPD) [182]. PEPD is a peptidase that is needed for IFNAR1 maturation and subsequent cell surface expression. NS5 binding of PEPD prevented maturation of complex N-linked oligosaccharides on IFNAR1, which in turn disrupted its surface expression (Figure 4B) [179,182]. In KFDV infection, IFN treatment failed to reduce viral titers when added after infection, this effect was also found to be mediated by the NS5 proteins antagonism [180,183].

During TBFV infection, a subgenomic noncoding RNA is formed, called subgenomic flavivirus RNA (sfRNA) [93,184,185]. It is produced as a product of incomplete degradation of genomic viral RNA by cellular 5′-3′ exoribonuclease XRN1 [184]. The ability to produce sfRNA in WNV was shown to be needed for efficient viral growth in vitro and for pathogenicity in mice [184]. Interestingly, DENV sfRNA was found to bind to TRIM25 to inhibit RIG-I-induced type I interferon expression in Huh-7 cells [186]. Furthermore, DENV and WNV sfRNA was found to suppress the RNAi response in both mammalian and insect cells [187]. Similarly, in TBEV infection, sfRNA has been demonstrated to inhibit the antiviral RNAi response in tick cells [93].

## 6. Future Considerations

Even though recent studies have shed light on the role of innate immunity during tick-borne flavivirus infection, much remains unknown. For example, the role of the TLR-TRIF and TLR-MyD88 pathway in pathogenesis and viral recognition. Furthermore, most studies were performed using LGTV and TBEV, and although they are closely related to POWV, LIV, KFDV, and OHFV, their interactions with the innate immune response might differ. Although IFN has been shown to strongly control viral tropism and pathogenesis of tick-borne flaviviruses, few antiviral ISGs have been identified. Viperin has been shown to be the most important ISG in cortical neurons, however, other ISGs that target TBEV expressed in astrocytes and granular cell neurons are yet to be identified and very little is known about the early response against the hemorrhagic TBFVs. We also know very little about how the innate immune response regulates the neuroinvasion of neurotropic TBFV, and the specific interactions between the tick vector and the different viruses.
